# Erratum: A biomimetic hybrid nanoplatform for encapsulation and precisely controlled delivery of theranostic agents

**DOI:** 10.1038/ncomms10350

**Published:** 2016-01-07

**Authors:** Hai Wang, Pranay Agarwal, Shuting Zhao, Jianhua Yu, Xiongbin Lu, Xiaoming He

Nature Communications 6: Article number: 1008110.1038/ncomms10081 (2015); Published: 12012015; Updated: 01072016

The original version of this Article contained an error in the spelling of ‘theranostic' in the title of the paper. This has now been corrected in both the PDF and HTML versions of the Article.

